# Ongoing slow oscillatory phase modulates speech intelligibility in cooperation with motor cortical activity

**DOI:** 10.1371/journal.pone.0183146

**Published:** 2017-08-11

**Authors:** Takayuki Onojima, Keiichi Kitajo, Hiroaki Mizuhara

**Affiliations:** 1 Graduate School of Informatics, Kyoto University, Sakyo-ku, Kyoto, Japan; 2 Rhythm-based Brain Information Processing Unit, RIKEN BSI – TOYOTA, Collaboration Center, RIKEN Brain Science Institute, Wako, Saitama, Japan; 3 Laboratory for Advanced Brain Signal Processing, RIKEN Brain Science Institute, Wako, Saitama, Japan; Baycrest Health Sciences, CANADA

## Abstract

Neural oscillation is attracting attention as an underlying mechanism for speech recognition. Speech intelligibility is enhanced by the synchronization of speech rhythms and slow neural oscillation, which is typically observed as human scalp electroencephalography (EEG). In addition to the effect of neural oscillation, it has been proposed that speech recognition is enhanced by the identification of a speaker’s motor signals, which are used for speech production. To verify the relationship between the effect of neural oscillation and motor cortical activity, we measured scalp EEG, and simultaneous EEG and functional magnetic resonance imaging (fMRI) during a speech recognition task in which participants were required to recognize spoken words embedded in noise sound. We proposed an index to quantitatively evaluate the EEG phase effect on behavioral performance. The results showed that the delta and theta EEG phase before speech inputs modulated the participant’s response time when conducting speech recognition tasks. The simultaneous EEG-fMRI experiment showed that slow EEG activity was correlated with motor cortical activity. These results suggested that the effect of the slow oscillatory phase was associated with the activity of the motor cortex during speech recognition.

## Introduction

Neural oscillations are attracting attention as underlying mechanisms of perceptual and cognitive abilities. Neural oscillation is a rhythmic neural activity that can be observed through EEG recordings. Recent studies have shown that cortical neural excitability depends on the phase of ongoing neural oscillations in sound detection tasks [1–3]. Neural excitability could modulate perceptual performance in visual or auditory processing [4–7]. Especially for auditory perception, the phase of slow oscillations (delta or theta activities) modulated behavioral performance in scalp EEG studies [4, 5]. Therefore, neural oscillation is an important activity that mediates the process of effective auditory perception.

The neural oscillatory phase can be modulated by periodic external input [1, 8]. In vocal communication, the speech rhythm entrained the neural activity in the listener’s brain. The delta and theta oscillations showed the phase synchronization with the speech envelope, inducing speech recognition [9]. The speech rhythm may shift the neural oscillatory phase in a listener’s brain to an optimal state in order to process the speech input. This indicates that the phase of slow oscillation also modulates speech recognition performance, as well as perceptual performance.

Neural oscillation might be associated with the enhancement of speech recognition when a listener looks at a speaker’s face [10]. When speech sound was distorted or embedded in noise, viewing a speaker’s face enhanced speech recognition [11]. The facial movement represented the timing of speech production and occurred before the speech production. Furthermore, the neural oscillatory phase before the sound presentation affected the behavioral performance in a sound detection task [4]. Therefore, it can be considered that the ongoing neural oscillatory phase contributes the prediction of speech production timing.

In addition, it is possible that viewing facial movements enhances the speech recognition with motor cortical activity. It is well known that the motor cortex contributes to speech perception [12–17]. In particular, phonetic identification depends on the motor cortical activity [12, 15–17]. A transcranial magnetic stimulation (TMS) on the motor cortex impaired phonemic identification in speech recognition [12, 16]. Furthermore, the activity of the motor system increased when viewing facial movements, as well as listening to speech [18]. The motor cortex might contribute to the enhancement of speech recognition by viewing the articulatory action.

Taken together, we hypothesized that the effect of the slow oscillatory phase was associated with the activity of the motor cortex in speech recognition. We assumed that the phase effect appeared before the speech presentation, as well as the sound detection. In addition to the phase effect, the motor cortex contributed to the enhancement of speech recognition. The activity of the motor system increased when viewing the articulatory action or listening to speech sound. The articulatory action occurred before the production of the speech sound and included the predictable information of the timing of speech production [10]. Therefore, we hypothesized that the phase effect was associated with motor activity.

To test our hypothesis, we measured human scalp EEG during a speech recognition task. We proposed an index to quantitatively evaluate the EEG phase effect on behavioral performance. By using the index, we showed that the phase of ongoing slow oscillation prior to the speech input modulated behavioral performance in speech recognition. The simultaneous EEG-fMRI analysis was then used to verify whether the motor cortical activity was related to the EEG activities during speech recognition.

## Materials and method

### Participants

Sixteen healthy participants (all right-handed, Edinburgh handedness test score 96.4±1.9 [mean±s.e.m.]; five female; mean age = 25 years with range of 21–32 years) engaged in the EEG experiment, and ten participants (all right-handed, Edinburgh handedness test score 98.0±2.0 [mean±s.e.m.]; two female; mean age = 30 years with range of 20–39 years) engaged in the simultaneous EEG-fMRI experiment. One participant was excluded from further analysis due to a low response rate below 60 percent in the EEG experiment. Another participant was excluded from further analysis due to an excessive artifact that could not be removed by EEG preprocessing in the EEG experiment. No participants were excluded in the simultaneous EEG-fMRI experiment. The experimental procedure was approved by the ethics committee of the Unit of the Integrated Studies of the Human Mind, Kyoto University (24-p-19). Participants gave their written informed consent according to the Declaration of Helsinki and were paid for their participation in the study.

### Experimental task

**Fig 1:**
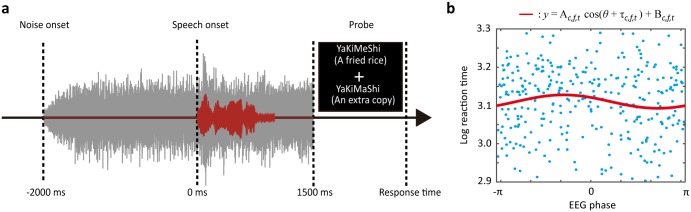
Schematic illustration of the experimental design and phase-utility on behavior (PUB) analysis. (a) Speech recognition task paradigm. Pink noise (shown as a gray wave in the figure) was presented after the trial interval, and a speech sound (shown as a red wave) was presented 2 sec after the onset of the noise. A probe was presented after the sound stimulus was presented. (b) An example of the relationship between the EEG phase and reaction time. The logarithmic reaction time was plotted as a function of the instantaneous EEG phase for each trial (blue plot). The red line was estimated by the least square method. The amplitude of the cos-curve was defined as the PUB index.

The experimental task was a speech recognition task with two alternative forced choices (Fig 1a). The participants were required to choose what they heard from one of the two choices (target and distractor) as soon and as accurately as possible. If the participants did not response within 2 sec, the trial was not used for further analysis. The auditory stimuli were four-letter Japanese words (e.g., *YaKiMeShi*, meaning fried rice), and were spoken within approximately 1 sec. The distractors were words in which either the second or third letter differed from the target (e.g., *YaKiMaShi*, meaning extra copy). The target and distractor were in the same range of word familiarity (5–6th on a scale of 1 [not familiar] to 7 [familiar]) [19]. A speech spectrograms were high-pass filtered with 3 Hz cutoff frequency in the EEG experiment to rule out the risk the neural entrainment through low-frequency component of speech rhythms (i.e., prosodic frequency [10]). The speech stimulus was then masked with pink noise. A signal-to-noise (SN) ratio of the speech stimulus was set to −10 dB in the EEG experiment. The SN ratio for the EEG experiment was decided to be approximately 80 percent of accuracy in the preliminary experiment with seven additional participants. Since MRI scans cause the big sound noise, the SN ratio of the simultaneous EEG-fMRI experiment was set to +5 dB to make the accuracies of speech recognition identical between the EEG experiment and the simultaneous EEG-fMRI. The accuracy was 73.9±1.5 percent (mean±s.e.m.) for the EEG experiment and 75.5±2.4 percent for the simultaneous EEG-fMRI. The reaction time was 1,190.0±44.2 ms for the EEG experiment and 1,510.0±46.9 ms for the simultaneous EEG-fMRI. The noise volume was gradually increased over 0.5 sec after the onset of the noise to avoid the EEG phase reset by the noise onset. The speech stimulus always started 2 sec after the noise onset and lasted approximately 1 sec. The speech stimulus was randomly chosen from 120 spoken words. The trials with 21 spoken words were discarded from further analysis due to low accuracy, which was below the chance level in the EEG experiment. The noise sound was terminated 1.5 sec after the onset of speech. The probe stimulus followed the speech stimulus and disappeared when the participants pressed any two keys. The reaction time was defined as the interval between the probe onset and the pressing of the key. The next stimulus started 1.167/1.262/1.357/1.452/1.547/1.642/1.737/1.833 sec after the termination of the probe stimulus. A white fixation cross was always presented at the center of the screen during the experiment. The EEG experiment consisted of four sessions, and the simultaneous EEG-fMRI experiment consisted of three sessions. Each session consisted of 100 trials.

### EEG recording

The EEG was recorded by a 32-channel EEG amplifier (BrainVision MR, Brain Products, Germany) with an international 10% standard electrode cap with a sintered Ag/AgCl ring electrode (Easy Cap, Falk Minow Services Germany). Four electrodes were used for the vertical and horizontal electrooculogram (VEOG and HEOG) channels. The measurement reference was linked earlobes, and the ground was on the inion. The sampling rate for the EEG was 5 kHz with a 1-Hz high-pass software filter, a 250-Hz low-pass hardware filter, and a 60-Hz notch filter. The impedance was set to below approximately 15 kΩ for all electrodes. All EEG experiments were done in a soundproofed room.

### Simultaneous EEG-fMRI recording

An MR-compatible amplifier (Brain Amp MR, Brain Products, Germany) was used to acquire the EEG during the fMRI measurements using a 10% standard system electrode cap with Ag/AgCl sensor electrodes (BrainCap MR, BrainProducts, Germany). The EEG cap had 27 EEG electrodes, one electrocardiogram (ECG) electrode, and four vertical and horizontal electrooculogram (VEOG and HEOG) electrodes. An FCz electrode, located between Fz and Cz, was used as the measurement reference, while an electrode on the inion was used as the measurement ground. The ECG electrode was set at the participant's back. The sampling rate for the EEG was 5 kHz with a 1-Hz high-pass software filter, a 250-Hz low-pass hardware filter, and a 60-Hz notch filter. The impedance was set to below approximately 15 kΩ for all electrodes. Blood oxygenation-sensitive echo-planar images (EPI) were obtained using a 3-tesla MR scanner (Magnetom Verio, Siemens, Germany) and were taken simultaneously with the EEG measurements. The participant’s head was immobilized using a vacuum pad during the measurements. The fMRI measurement settings were as follows: repetition time (TR) = 2 sec, echo time (TE) = 30 msec, flip angle (FA) = 80 deg, field of view (FoV) = 192 mm, matrix size = 64 × 64, slice thickness = 5 mm, gap = 0 mm, 333 volumes, and 3 sessions. Prior to wearing the EEG cap, a structure image was acquired for all the participants through a magnetization prepared rapid gradient echo (MPRAGE) with the following settings: TR = 2.25 sec, TE = 3.06 msec, FA = 9 deg, FoV = 256 mm, matrix size = 256×256, slice thickness = 1 mm, gap = 0 mm.

### EEG preprocessing

Ocular artifacts in the EEG were corrected using an EEG analysis software (Brain Vision Analyzer, Brain Products, Germany), by subtracting the voltages of the VEOG and HEOG channels, multiplied by a channel-dependent correction factor calculated by linear regression among the VEOG/HEOG and EEG electrodes [20]. The reference for the ocular-corrected EEG was then changed to the average of all electrodes, except the VEOG and HEOG. MR-scan-related and ECG-related pulse artifacts were removed using EEG analysis software (Brain Vision Analyzer, Brain Products, Germany) before the ocular artifact correction for the EEG data that was recorded simultaneously with the fMRI data. Briefly, the waveforms for the MR-scan-related and ECG-related artifacts were identified by averaging the EEG data derived from the artifact periods and were subtracted from the EEG data [21, 22]. The preprocessed EEG data were then downsampled to 500 Hz in the EEG experiment data and 200 Hz in the data from the simultaneous EEG-fMRI experiment and then exported to Matlab (Mathworks Inc., USA). The EEG data were transformed into time-frequency representations of EEG power and phased from 1 to 60 Hz in 50 logarithmically spaced steps, using the Morlet wavelet. The mother wavelet was defined by the following equation [23]:

$$\text{w}\left(t,f\right)={\left(\sigma_{t}\sqrt{\pi }\right)}^{-\frac{1}{2}}\text{exp}\left(\frac{-t^{2}}{2\sigma_{t}^{2}}\right)\text{exp}\left(\text{i}2\pi ft\right),$$ (1)

where *t* is time, *f* is frequency, and *σ*_*f*_ is the variance at the frequency *f*. The variance *σ*_*f*_ is expressed by *σ*_*t*_, which is the variance at time *t*, as *σ*_*f*_ = 1/(2π*σ*_*t*_). The wavelet is characterized by the ratio of the frequency to variance (*f*/*σ*_*f*_) and was set to (*f*/*σ*_*f*_) = 7 in the current study. By convolving the mother wavelet (Eq 1) to the EEG time series *s*_*c*_(*t*), the EEG power *p*_*c*_(*t*, *f*) and phase θ_*c*_(*t*, *f*) at electrode *c* were computed by the following equation:

$${\text{p}}_{c}\left(t,f\right)={\text{ log}}_{10}\left({\left|\text{w}\left(t,f\right)*s_{c}\left(t\right)\right|}^{2}\right),$$ (2)

$${\text{e}}^{\text{i}\theta_{c}\left(t,f\right)}=\text{ w}\left(t,f\right)*{\text{s}}_{c}\left(t\right)/\left|\text{w}\left(t,f\right)*s_{c}\left(t\right)\right|.$$ (3)

The * indicates the convolution operator. The time series *s*_*c*_(*t*) was the segmented period from 2 sec before to 1.5 sec after the speech onset for the EEG analysis, while it contained the whole length of the EEG data in each session for the simultaneous EEG-fMRI analysis. Because of the computational problem, the EEG power and phase were downsampled into 100 Hz for phase-utility on behavior (PUB) computation.

### EEG analysis

#### Phase-utility on behavior

We proposed the PUB index to evaluate how much behavioral performance depends on the ongoing EEG phase. Behavioral performance (accuracy or reaction time) were sorted by instantaneous phase and fitted by the cos-curve with the least square method (Fig 1b). The accuracy was derived from the segmentation with eight bins of EEG phase (*y*_*i*_, *i* = 1, 2, ⋯, 8), and the reaction time was transformed into logarithmic scale (*y*_*i*_ = log_10_ [RT(*ms*)], *i* = 1, 2, ⋯, #trials). The independent variable was the instantaneous EEG phase (*θ*_*i*_, *i* = 1, 2, ⋯, #trials) in the least square method. Thus, the computation of the PUB was defined by the following equation:

$$y={\text{A}}_{c}\left(t,f\right)\text{cos}\left(\theta_{c}\left(t,f\right)+\tau_{c}\left(t,f\right)\right)+{\text{B}}_{c}\left(t,f\right)+\xi_{c}\left(t,f\right),$$ (4)

where *c* is the electrode, *t* is time, *f* is frequency, A is the regression coefficient, B is the intercept, and *τ* is the parameter of shifting the cos function. The constant τ (0 ≤ τ ≤ π) was decided to minimize the sum of residuals ***ξ***. The amplitude |A| is defined as the PUB at the optimal τ. A larger PUB means a larger dependence of behavioral performance on the EEG phase and vice versa. Based on the constant τ, we can compute the optimal and non-optimal phase showing the best and worst behavioral performance. When A was a positive value, the optimal phase was π − τ. When A was negative value, the optimal phase was − τ. Note that the index could be computed appropriately only when the phase was uniformly distributed among the trials, since the dependent variable must be explained by the phases ranging from 0 to 2π in the least square method. If the EEG phase was locked among the trials, it decreased the accuracy of the PUB computation. We computed the PUB by using all trials except those in which the participants failed to press a key within 2 sec. The number of remaining trials was approximately 305.9±7.6 (mean±s.e.m. among fourteen participants). When we computed the PUB using only the correct trials, the results were similar to the phase-dependent behavioral modulation that was computed using all the trials (S1 Fig). Therefore, we decided not to exclude the incorrect trials in order to get better statistical power for the PUB computation, which required larger numbers of trials for the analysis.

#### Statistical test

**Fig 2:**
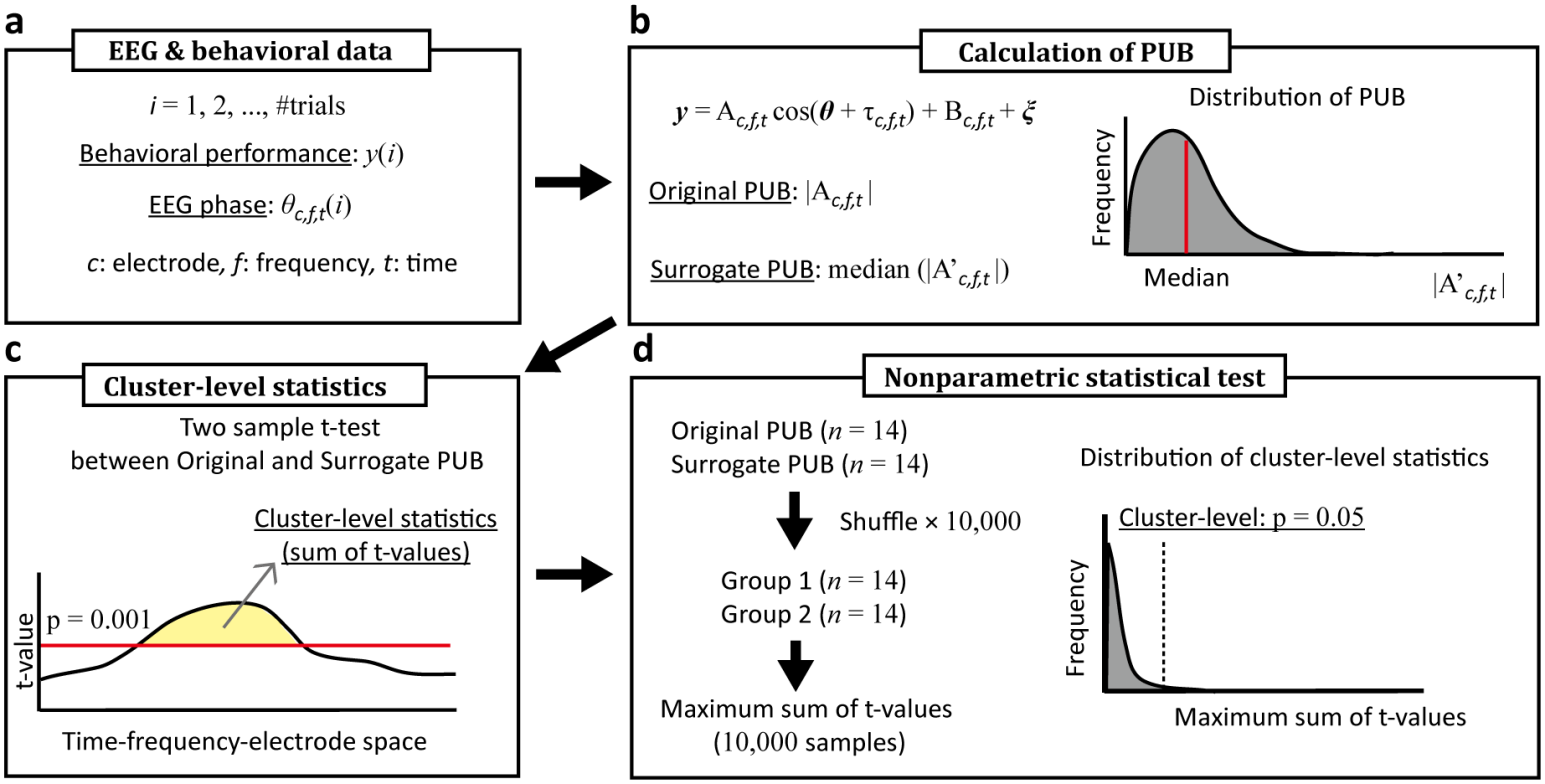
Schematic illustration of the cluster-level statistical test. (a) The EEG was decomposed into instantaneous phases. (b) The original PUB and surrogate PUB were calculated from the EEG phases and behavioral performance data. (c) Clusters in the time-frequency-electrode space were decided based on the threshold of the t-value, and the cluster-level statistics were computed. (d) The cluster-level statistics were tested by the empirical distribution of the maximum cluster-level statistics.

For the statistical analysis, the t-test between the PUB and surrogate data was used to test whether the behavior performance depended on the EEG phase. The surrogate data for the PUB analysis were computed using the random sampling data of the behavioral performance instead of the performance vector **y** in Eq (4) (Fig 2a and 2b). The random sampling was repeatedly computed 200 times, and the median values of these data were used as the surrogate data for each participant. The distribution of these data were non-normal and asymmetrical in form, since the PUB was computed as the absolute value, leading us to use the median of the PUB distribution for the surrogate data. The t-values were calculated by two sample t-tests between the original PUB and surrogate PUB. To avoid the multiple comparison problem, the statistical threshold was decided by a nonparametric statistical test derived from a cluster-based Monte Carlo p-value [24]. The PUB clusters were decided by the temporal, spectral, and spatial adjacency of the samples that exceeded a student’s t-value (p < 0.001) (Fig 2c). The cluster-level statistics were the summation of the t-values within a cluster. To decide the cluster-level threshold, the original PUB and surrogate PUB were pooled into one group and randomly divided into two groups. Then, the cluster-level statistics were decided in the same manner as above (Fig 2d). The random shuffling of the original PUB and surrogate PUB was repeated 10,000 times, and the maximum cluster-level statistic was computed to obtain the empirical distribution. The statistical threshold was decided by the empirical distribution of the maximum cluster-level statistics (p < 0.05). We excluded clusters showing no spatial expansion, and the extent of the threshold was < 2 electrodes.

**Fig 3:**
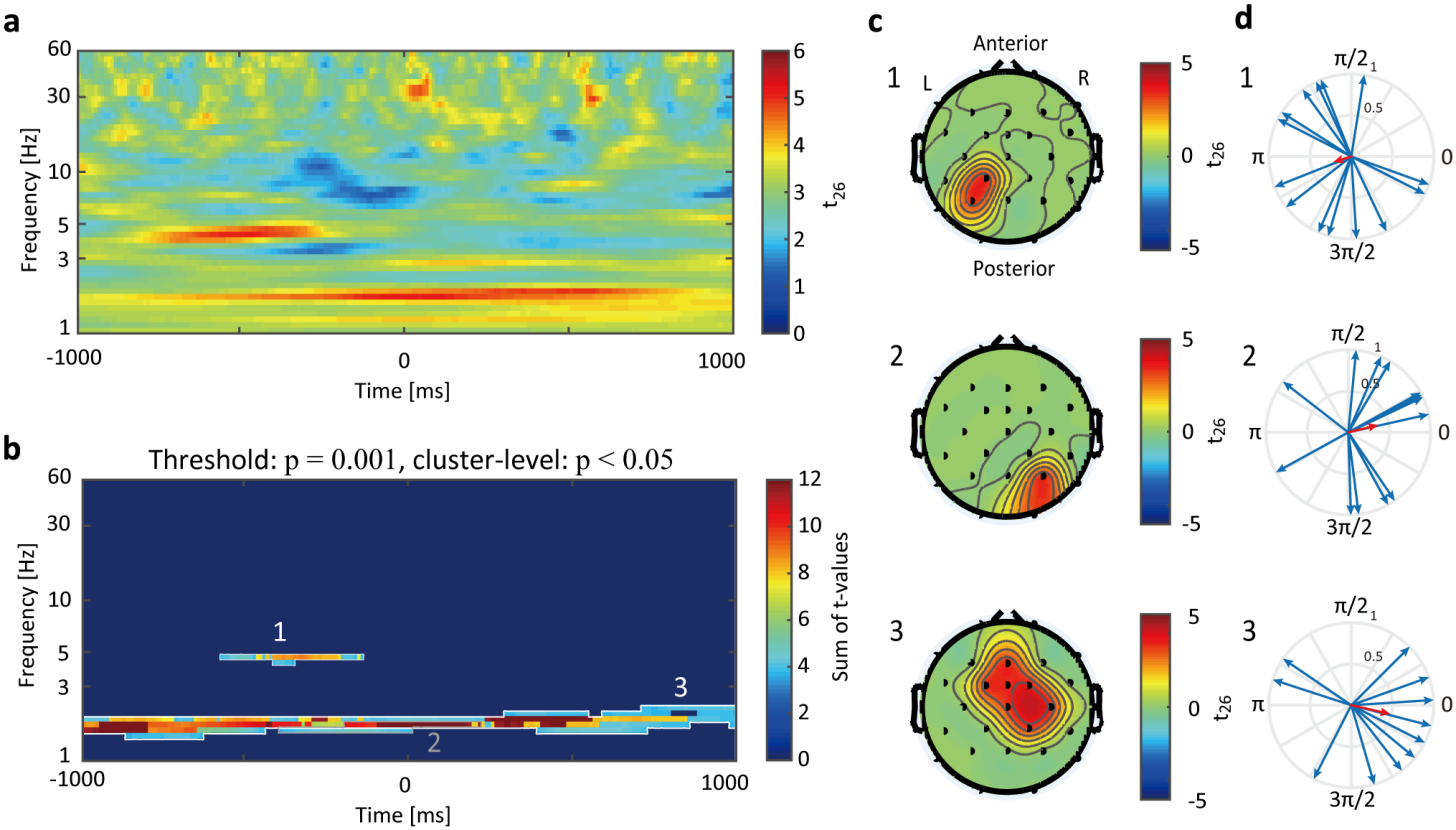
Phase-utility on behavior (PUB) result. (a) Statistical t-map of the comparison between averaged PUB across all electrodes and surrogate data. The color bar represents the t-value of the two-sample t-test (n = 14). Larger t-values mean the PUB increased relative to the surrogate. (b) Cluster-level analysis of the PUB and surrogate comparison based on the temporal, spectral, and electrode adjacency. The three areas indicated by the numbers 1–3 in the figure are the significant clusters (cluster level p < 0.05). For presentation purposes, the color bar represents the summation of t-values among the electrodes, while the significant clusters were decided through a summation of the temporal, spectral, and electrodes spaces. (c) The scalp topographies show the average t-values among the temporal and spectral spaces of each cluster. The numbers 1–3 corresponded to the cluster numbers in (b). (d) Individual optimal phase (blue arrows) and circular mean among all participants (red arrow; top: CP1 theta; middle: P4 delta; bottom: FC2 delta). The position of time-frequency electrode was chosen to the show maximal PUB value within each cluster for this circular mean analysis. The Rayleigh test for uniformity was applied in each of the optimal phases (CP1 theta, *Z*(14) = 0.7207, p > 0.05; P4 delta, *Z*(14) = 1.9491, p > 0.05; FC2 delta, *Z*(14) = 3.2771, p < 0.05).

In order to confirm the effect of the EEG phase, we used an ANOVA analysis to compare the reaction times between the trials of the optimal and non-optimal phases. There is a risk of double dipping when the data point of the EEG phase is simply decided by the t-value, which is calculated with all the participants. To avoid the double dipping problem, we used the leave-one-participant-out cross-validation procedure, which gave a data point for each participant that could be used in the ANOVA [25]. The t-values were calculated by a two-sample t-test among N − 1 participants, where N was the number of participants. The data point of the EEG phase for one participant was, thus, decided by the t-value from the remaining participants. The t-values calculated with other participants were independent from the data point for one participant. The clusters were then decided by the temporal, spectral, and spatial adjacency of the samples that exceeded the t-value (p < 0.01). The clusters corresponding to the clusters in Fig 3 were decided by eye inspection for each participant. If the clusters derived from the leave-one-participant-out procedure were not isolated from the other clusters, the clusters were manually separated based on the time-frequency-electrode space between the clusters in Fig 3. We used the data points that had the maximum t-value in each cluster for the individual participant. The trials were classified into six groups based on the estimated optimal phase by the PUB analysis. The averaged logarithmic reaction times from the trials in the EEG phase groups were compared each other using an ANOVA analysis.

### Simultaneous EEG-fMRI analysis

SPM12 software (Wellcome Department of Cognitive Neurology, London, UK, URL:www.fil.ion.ucl.ac.uk/spm) was used for the image preprocessing and voxel-based statistical analysis of the fMRI data. The first three volumes were discarded, and slice timing was corrected on the middle slice to remove the time delay from scanning the entire brain. The echo-planar images (EPIs; 330 volumes for each session × 3 sessions) were transferred into the first image volume for each participant to correct for head motion. The individual EPIs were normalized to a standard brain by applying the parameters estimated by matching the MPRAGE anatomical image to the stereotactic coordinate image from the Montreal Neurological Institute. The EPIs were then smoothed with a 10-mm full-width half-maximum Gaussian kernel. The following general linear model was used to identify the cortical areas that correlated with the EEG activity:

Y=Xβ+ε, (5)

where **X** is the design matrix consisting of the EEG power, task-related model, and the six head-motion parameters, **Y** is the vector of fMRI BOLD signals, **β** is the regression coefficient vector, and ***ε*** is the residual vector. The design matrix for the EEG activity was computed by the convolution of the canonical hemodynamic response function and the EEG power time series to bridge the temporal gap between the fMRI and EEG data, and then subsampled at the time of scanning the fMRI measurements [26–28]. The EEG power time series was z-transformed before the convolution of it with the hemodynamic response function. The task-related model was computed by convolving the hemodynamics response function with the probe presentation duration to reduce the effect of the visual presentation. The regression coefficients for the EEG powers were computed for each participant using the fixed effect model, and then taken into the group analysis using a random-effect model of a one-sample t-test.

## Results

### Changing behavioral performance based on EEG phase

**Fig 4:**
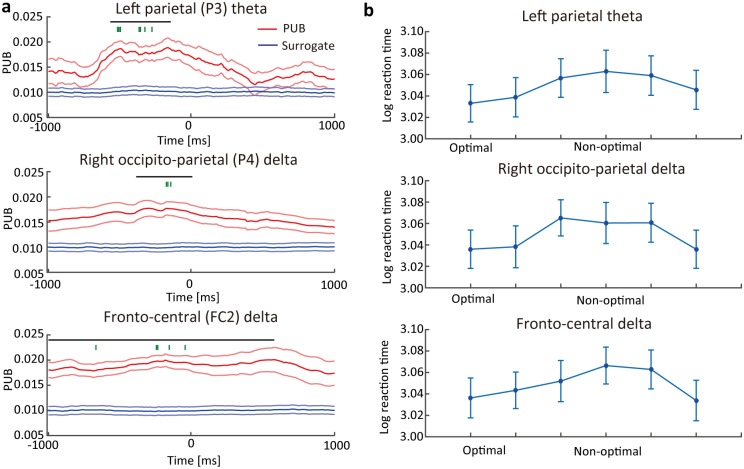
The effect of the phase on reaction time. (a) The PUB (red line with ± s.e.m.) and surrogate value (blue line ± s.e.m.) were averaged among the participants for the P3 (top), P4 (middle), and FC2 (bottom) electrodes. The black horizontal bars on each panel represent the period when a significant difference occurred (cluster level p < 0.05). Green vertical bars indicate the time point for the ANOVA analysis selected by the leave-one-participant-out cross-validation statistics. (b) Logarithmic reaction time as a function of phase bins derived from the PUB analysis. The trials were classified into six groups based on the optimal phase estimated by the PUB analysis. The blue plots and vertical bars represent the average log-reaction times and their s.e.m.

To test whether behavioral performance in speech recognition changed based on the ongoing EEG phase, we evaluated the relationship between the accuracy, reaction time and EEG phase by using the proposed PUB index. The PUB analysis was applied to the EEG experiment but not to the simultaneous EEG-fMRI analysis because the EEG data from the simultaneous recording was insufficient to analyze the details of the EEG phase due to the MRI-related artifacts. The delta and theta activities showed significant PUB modulation in the reaction time analysis (Figs 3 and 4). The comparison of the grand averaged PUB and surrogate showed theta activity (4–5 Hz) just before the speech presentation and delta activity (1–2 Hz) around the onset of speech (Fig 3a). We confirmed that these activities were statistically significant using a cluster-level test that was based on temporal, spectral, and electrode adjacency (cluster level p < 0.05; Fig 3b). The topographies showed that theta activity appeared around the left parietal electrodes (P3 and CP1) (Fig 3c-1). The delta activity was spatially divided into two clusters: right occipito-parietal electrodes (P4 and O2) (Fig 3c-2), and fronto-central electrodes (Fz, Cz, FC1, FC2, C4, CP2 and FCz) (Fig 3c-3). To confirm the consistency of the optimal phase among the participants, a selected point showed maximum PUB value within each cluster, and the phase consistency at this point was verified by the Rayleigh test (Fig 3d). The results showed significant phase consistency on the fronto-central delta oscillation (p < 0.05) (Fig 3d-3). The left parietal theta and occipito-parietal delta activities were not consistency among all participants (p > 0.05) (Fig 3d-1 and 3d-2). The accuracy analysis showed no statistically significant results (cluster level p > 0.05). Therefore, further analyses focused on the reaction time.

To confirm whether the optimal/non-optimal phase derived from the PUB analysis showed a behavioral difference, we compared the reaction times between the optimal and non-optimal phases. To avoid double dipping, the data point of the time-frequency-electrode space for the comparison was individually decided using the leave-one-participant-out cross-validation procedure [25]. The data points were the maximum t-value in the space of each cluster in each leave-one-participant-out group for each participant (Fig 4a). Based on the optimal/non-optimal phase, we classified all trials into the six bins. The ANOVA analysis showed the main effect of the reaction time among these bins (left parietal theta, *F*(5,65) = 6.31, p < 0.001; right occipito-parietal delta, *F*(5,65) = 12.198, p < 0.001; fronto-central delta, *F*(5,65) = 10.538, p < 0.001) (Fig 4b). The results indicate that our method can successfully identify the EEG activity whose phase modulated the reaction times.

### Brain areas involved in PUB activity

**Fig 5:**
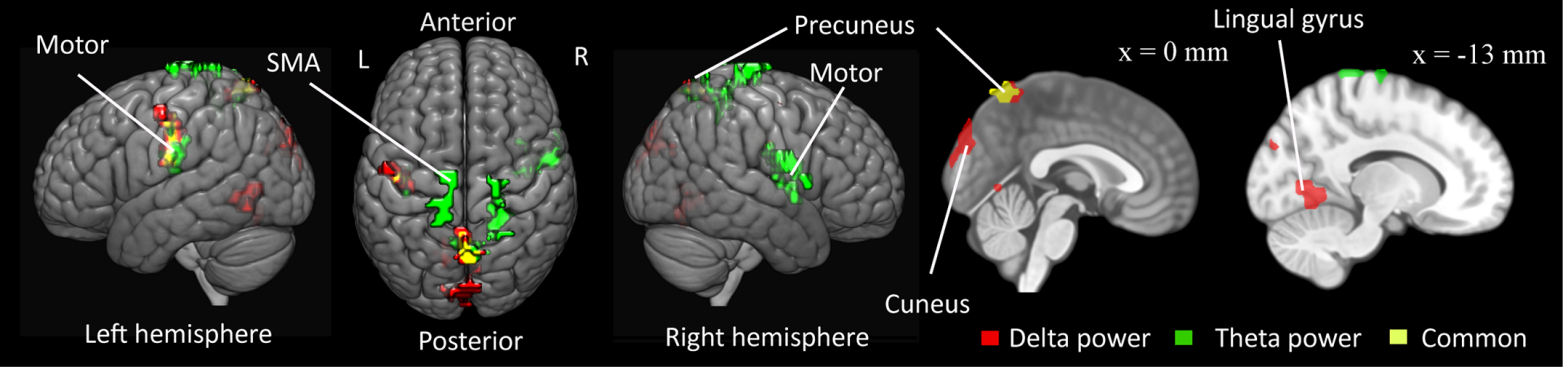
Brain areas for EEG activity. The brain areas showing correlations with the left parietal theta and fronto-central delta power fluctuation. The red areas indicate a significant correlation between the delta power and the BOLD time series (cluster-level FWE-corrected p < 0.05). The green areas indicate a significant correlation with theta power. The yellow areas indicate common areas that showed correlations with delta and theta power.

We performed a simultaneous EEG-fMRI analysis to identify the brain areas associated with the EEG that also showed PUB modulation. The EEG and fMRI were combined through a multiple regression analysis. The EEG regressor, which was simultaneously recorded with the fMRI, was composed of a power of theta or delta oscillations whose electrode and frequency were decided based on the PUB results (see Fig 3c; the left parietal theta regressor was the mean theta power between the P3 and CP1 electrodes; the right occipito-parietal delta regressor was the mean delta power between the P4 and O2 electrodes; the fronto-central delta regressor was the mean delta power among the Cz, FC1, FC2, C4, CP2, and FCz electrodes). We performed a voxel-based statistical analysis of the fMRI BOLD images to identify the cortical areas of the theta and delta bands activities (cluster-level FWE-corrected p < 0.05). The left parietal theta regressor was computed by averaging the EEG powers of the P3 and CP1 electrodes at a frequency of ~4.5Hz (Fig 3c-1). The theta power was positively correlated with the activities in the bilateral motor cortices, supplementary motor area (SMA), and precuneus (Table 1; Fig 5). The fronto-central delta regressor was computed by averaging the EEG power of the Fz, Cz, FC1, FC2, C4, and CP2 electrodes at a frequency of ~1.7Hz (Fig 3c-3). The delta power was positively correlated with the activities in the left motor cortex, cuneus, precuneous, and lingual gyrus (Table 1; Fig 5). The left-parietal delta regressor was derived from the P4 and O2 electrodes at a frequence of ~1.5 Hz (Fig 3c-2) and showed no significant response.

**Table 1:** Brain areas showing correlations with EEG activities.

EEG activity	Anatomical region	MNI coordinates (mm)			t-value
		X	y	z	
Left parietal theta	l-Motor (BA4)	-39	-13	25	8.09
	r-Motor (BA4)	57	-1	20	9.15
	r-Precuneus (BA7)	6	-69	65	8.12
	r-SMA (BA5)	18	-49	65	7.34
	l-SMA (BA6)	-15	-16	80	7.07
Right occipito-parietal delta	None				
Fronto-central delta	l-Motor (BA4)	-45	-10	50	9.86
	Lingual gyrus (BA19)	-12	-70	5	9.77
	Cuneus (BA10/9)	0	-97	20	7.67
	r-Precuneus (BA7)	3	-67	65	5.89

(SMA: supplementary motor area; BA: Brodmann area; MNI: Montreal Neurological Institute; l-: left; r-: right)

## Discussion

### Delta and theta oscillations in speech recognition

We evaluated the correlation between the phase of ongoing EEG activity and behavioral performance using the PUB analysis. The ongoing EEG phase modulated neural excitability and perceptual performance during the visual/auditory perception tasks, even if it appeared prior to the stimulus onset [1, 4, 6]. In line with previous studies, we successfully found that the EEG phase accompanied behavioral modulation during a speech recognition task. The PUB index can continuously evaluate the phase-dependency of behavioral performance, while previous methods evaluate this dependency discretely [6]. In addition to the previous method, the circular mean can compute a similar PUB value where the reaction time and EEG phase are defined as the length and angle of the vectors, respectively. When distribution of the EEG phase is uniform, the length of circular mean vector indicates the phase-dependency of the reaction time. However, the length of the circular mean vector increases irrespective of behavioral performance when the EEG phase is locked (S2 Fig). In contrast, the PUB value is close to zero in the phase-locking condition. However, the PUB also has a problem: the mean value depends on the numbers of trials, as well as the circular mean. Therefore, it is necessary to compare the original PUB to surrogate the PUB to avoid this problem. The number of trials with the surrogate PUB was the same as the original PUB. Therefore, the comparison can identify the phase-dependency of the behavior performance without considering the number of trials. This might enable a more sensitive identification of the phase and behavior relationship compared to the previous method and the circular mean. Our PUB result demonstrated that the reaction time was modulated depending on the theta phase prior to speech onset. It was also reported that the theta oscillatory phase affected the behavioral performance in a sound detection task [4]. Our PUB method clearly found that the ongoing theta oscillation was correlated with the performance of speech recognition. This implied that the influence of the theta phase appeared not only in perceptual performance, but also in recognition performance.

In addition to the theta oscillation, we found that the delta oscillation modulated the reaction time. The front-central delta activity was the only oscillation that showed a consistent optimal phase among all participants (Fig 3d-3). The delta and theta oscillations are known to be crucial components of vocal communication [10, 29, 30]. The speech sound has prosodic and syllabic rhythms. The prosody is the stress and intonation patterns of an utterance. The envelope of prosodic rhythms appears in the 1–3Hz frequency range. The syllable is a unit of speech that separates a word into sound chunks. For example, the word “oscillation” is composed of four syllables: “os-cil-la-tion” and its sound envelope appears in the 4–8 Hz frequency range in vocal communication. The frequency of the delta oscillation is identical to the frequency of the prosodic rhythms, and the theta oscillation corresponds to the syllabic rhythms. These speech rhythms were synchronized with the listener’s EEG oscillations in the delta or theta frequency [9, 30]. The synchronization was stronger when the speech was recognized, in contrast to when the speech was presented backward. Together, our findings on delta and theta activities are consistent with previous studies showing that these slow EEG activities is associated with the recognition of speech.

### Motor contribution in speech recognition

We showed that the delta and theta EEG phases contributed not only to sound detection performance, but also to speech recognition performance. We then asked how the EEG phase affects behavior. The fronto-central delta and left parietal theta powers were correlated with the BOLD activities in the motor cortices in the simultaneous EEG-fMRI experiment. However, the scalp distribution of the EEG oscillations was seemingly unrelated to the cortical areas. In the simultaneous EEG-fMRI analysis, we computed the correlation between EEG power fluctuation and BOLD fluctuation. If a cortical activity showed a high temporal correlation with EEG power, it was considered part of the related cortices. In this case, the results included not only the source (generator) of the scalp-EEG activity, but also activities that had temporal correlations with the real source. Therefore, seemingly unrelated cortical activity may be detected if it shows a temporal correlation with the scalp-EEG power. The results from the simultaneous EEG-fMRI experiment didn’t allow us to determine what cortex was the real “source” of scalp EEG activity. In this case, we were able to consider that the cortical activities—those appeared in the results from the simultaneous EEG-fMRI experiment—were “associated” with the scalp EEG-activity. Our result showed motor activity was clearly found in the results from the simultaneous EEG-fMRI analysis, which was consistent with a previous study [31]. The results provided important information on the functional relevance of fronto-central delta and left parietal theta oscillation in cooperating with the motor cortex.

Motor cortical activity contributes to speech perception (e.g., motor theory of speech perception) [13, 14, 32, 33]. Speech intelligibility is enhanced when the listener can identify the speaker’s motor signals for speech production. The muscle activity for lip movement increases when viewing a speaker’s face or listening to speech sounds [18]. The neural activities in the motor cortex for the lip and tongue movement also enhanced speech comprehension in a previous TMS study [12]. The activity of the motor cortex particularly affects phonemic identification when listening to speech sounds with noise [12, 15–17]. The motor cortex is not necessary for speech recognition in general, but it may help to compensate for distorted speech sounds [34]. Speech stimuli were also embedded in noise sound during our EEG and simultaneous EEG-fMRI experiments. Under these experimental conditions, it was necessary to compensate for the degraded speech sound by viewing the speaker’s facial movements. Together with these previous studies, our simultaneous EEG-fMRI experiment supported the contribution of motor cortex activity in speech recognition. The spatial location of motor activity in the results from the simultaneous EEG-fMRI experiment was consistent with the spatial coordinates for lip movement [35]. This suggested that the delta and theta activities corresponded with the activity in the motor cortex that controlled lip movement.

The delta and theta oscillation in the motor cortex plays a role in top-down control for auditory activity in continuous speech recognition [31]. The auditory delta and theta oscillations were phase-synchronized with the rhythms of the speech envelope during vocal communication. The top-down signal increased the synchronization between the neural oscillation and the speech envelope to enhance speech intelligibility. This implies that the delta and theta oscillations in the motor cortex are a crucial component for enhancing speech recognition. The finding of the delta and theta phases in the current study is in line with the previous study [31]. Therefore, the delta and theta phases in the PUB analysis might be involved in the top-down signal for speech recognition.

One may be concerned with the possibility of motor activity for finger movements in association with the participants’ response. In fact, the theta activity was related to the activity in the SMA during a motor executive task [36]. We found theta power modulation that was associated with the participants’ responses in the current study (data not shown). The motor activity enhanced the movement speed in cooperation with the distributed brain activities via delta synchronization [37]. In our results, the delta oscillation was, in fact, accompanied by activity in the motor cortex along with activities in the cuneus, precuneus, and lingual gyrus. These results may raise the risk that shorter reaction time was merely induced by speeding up the participants’ response. However, the spatial location of the motor activity was found in the region that controls lip movement but not finger movement. In addition to the location of motor activity, the paramidline theta activity was related to speech recognition[31]. Taken together, our results supported the idea that motor activity was not only related to the listener’s finger response but also with speech recognition, while the possibility of contamination from the finger response could not be completely denied in the activity in the motor cortex and SMA.

Another concern is whether the EEG activities were identical between the EEG experiment and the simultaneous EEG-fMRI experiment. The electrode position and frequency of the simultaneous EEG-fMRI analysis was decided based on the results of the EEG experiment without fMRI. Here, we assumed that EEG activities (those used for the simultaneous EEG-fMRI analysis) were identical to the activities showing the phase-dependency of the behavioral modulation in the EEG experiment. The accuracies were identical; however, the noise level was different between these two experiments. The temporal profile of the EEG power modulation was also identical when associated with the fronto-central and occipito-parietal delta activities (middle and right figures of S3 Fig). Note that the left parietal theta activity showed a different temporal profile of EEG power modulation for the EEG experiment and the simultaneous EEG-fMRI experiment (left figure of S3 Fig; p < 0.05), which might have been caused by the different experimental environments. Noise always occurred inside the MRI scanner during the MRI recording; however, there was no sound input in between the trials during the EEG experiment. The theta activity was an important component for sound input and showed the modulation of EEG power caused by the MRI noise [38]. The MRI noise (before the noise onset in left figure of S3 Fig) might have modulated the theta EEG power, resulting in a different temporal profile of the power at the left parietal electrode between the EEG experiment and the simultaneous EEG-fMRI experiment. Therefore, the different temporal modulation of the theta EEG power did not disprove our assumption that the activity in the simultaneous EEG-fMRI experiment was identical to the activity during the EEG experiment. These results strongly supported our assumption that the results of cortical activities in the simultaneous EEG-fMRI analysis were tightly correlated with the delta activities, which showed the phase dependent behavior modulation. Meanwhile, further research is needed to determine the results related to theta activity.

### Predicting sound input

Our results suggested that speech recognition was enhanced when a participant could identify the motor activity of another person’s speech through delta and theta oscillation. On the other hand, effects of the delta and theta phases not only appeared in speech recognition, but also during simple tone detection [4, 5]. This raises the question of whether the phase of delta and theta oscillations identified the speaker’s speech production in speech recognition, or purely predicted the auditory input timing in sound detection. One possible interpretation of our results is that the selective attention in time for sensory inputs enhanced the performance of speech recognition with low-frequency oscillations [10].

In sensory perception, low-frequency oscillations were associated with selective attention or prediction for external sensory inputs [4, 5, 39, 40]. Furthermore, a previous study reported that this prediction was realized by the neural synchronization in the motor cortex [41]. In speech recognition, the delta and theta oscillations were also considered a predictive function for speech input. Speech recognition can be enhanced by predicting the timing of speech input by viewing the speaker’s face [10]. The speaker’s facial movements coincide with the prosodic rhythm in speech, and the frequency of prosody is known to appear in the delta range (1–3Hz). In addition to the delta oscillation effect in speech recognition, the theta oscillation coded the visual feature, particularly in terms of mouth shape [42]. The theta oscillation is also an important rhythm in speech processing (i.e., syllabic rhythm) [29]. These delta and theta oscillations may be synchronized with head and mouth movements, resulting in a prediction of the auditory input to enhance speech recognition [10]. Therefore, the delta and theta oscillations in the current study might be associated with predicting the auditory input timing, thus enhancing speech intelligibility.

Another possible interpretation of the behavioral modulation by the delta or theta oscillatory phases is articulatory prediction by the motor cortex. Neural excitability could be enhanced during an optimal phase and suppressed during a non-optimal phase of the slow neural oscillation. Our simultaneous EEG-fMRI experiment showed that slow neural oscillation was related to activity in the motor cortex. This motor activity is related to recognition at the phonemic level and generates articulatory prediction during the phoneme discrimination task [12, 15–17, 34]. The target and distractor words differed only at one phonemic level in this study. The phonemic discrimination might be accelerated by enhancing the neural excitability in the motor cortex for tongue or lip motion through the effect of phase. The neural mechanism of behavioral modulation could be predicted by the slow neural oscillation; however, future research will need to determine whether this prediction acts on the timing or articulation of speech sounds.

Our study provided a new index to quantitatively evaluate the EEG phase effect on behavior performance and indicated that the delta and theta phases modulated reaction times in speech recognition. The simultaneous EEG-fMRI analysis showed that motor cortical activity was correlated with the delta and theta oscillations. These results suggested that the phase-dependency of speech intelligibility was associated with the contribution of the motor cortex for speech recognition.

## Supporting information

S1 Fig PUB value of all trials and correct trials. The PUB values of all trials (left) and correct trials (right) were averaged among all electrodes and all participants.
(TIF)

S2 FigPUB values and absolute values of the circular mean.The PUB values (left) and absolute values of the circular mean (right) were averaged among all the trials for the CP1 electrode. The circular mean was defined as $\frac{1}{\text{N}}\sum_{i}^{\text{N}}r_{i}{\text{e}}^{\text{i}\theta_{i}}$, where the *r*_*i*_ is the logarithm reaction time of *i* trial and *θ*_*i*_ is the EEG phase. The logarithm reaction time was scaled from 0 to 1. The absolute value of the circular mean indicated the phase-dependency of the reaction time, as well as phase-locking among all the trials.(TIF)

S3 Fig EEG power modulation associated with the onset of noise and speech. The EEG power for the EEG experiment (blue line) and the simultaneous EEG-fMRI experiment (red line). The power was averaged among the participants (mean±s.e.m.) for the left parietal theta (left), right occipito-parietal delta (middle), and fronto-central delta (right). The black horizontal bars on each panel represent the period where the significant difference was shown (p < 0.05).
(TIF)

S1 File PUB data. The PUB data includes participants’ original PUB and surrogate PUB data.
(ZIP)

S2 File Contrast in SPM. SPM contrast for fMRI activations.
(ZIP)
