# Supplementary figures and images for: Ongoing slow oscillatory phase modulates speech intelligibility in cooperation with motor cortical activity
Source: PLoS One. 2017 Aug 11;12(8):e0183146. doi: 10.1371/journal.pone.0183146 (PMC5554004; doi:10.1371/journal.pone.0183146)

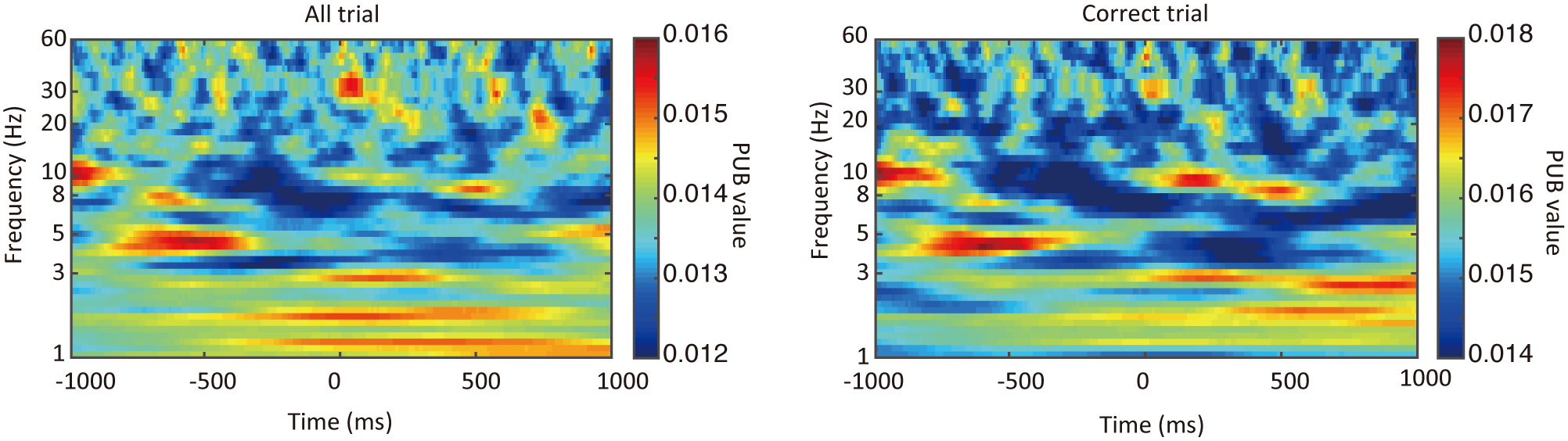

Supplement: S1 Fig — The PUB values of all trials (left) and correct trials (right) were averaged among all electrodes and all participants. (TIF) [file pone.0183146.s001.tif]

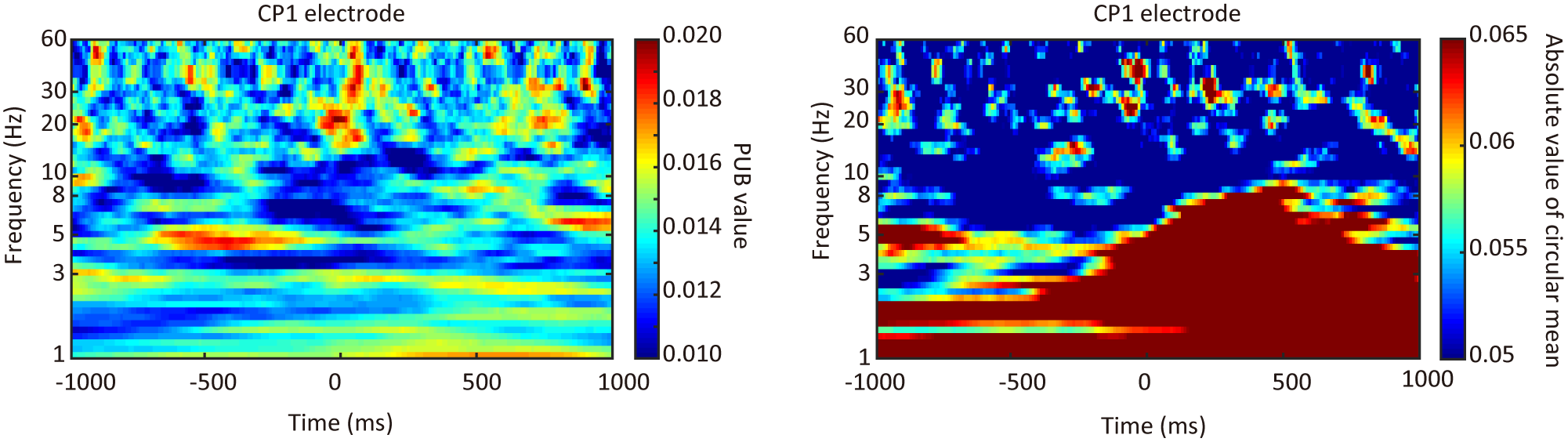

Supplement: S2 Fig — The PUB values (left) and absolute values of the circular mean (right) were averaged among all the trials for the CP1 electrode. The circular mean was defined as 1N∑iNrieiθi, where the ri is the logarithm reaction time of i trial and θi is the EEG phase. The logarithm reaction time was scaled from 0 to 1. The absolute value of the circular mean indicated the phase-dependency of the reaction time, as well as phase-locking among all the trials. (TIF) [file pone.0183146.s002.tif]

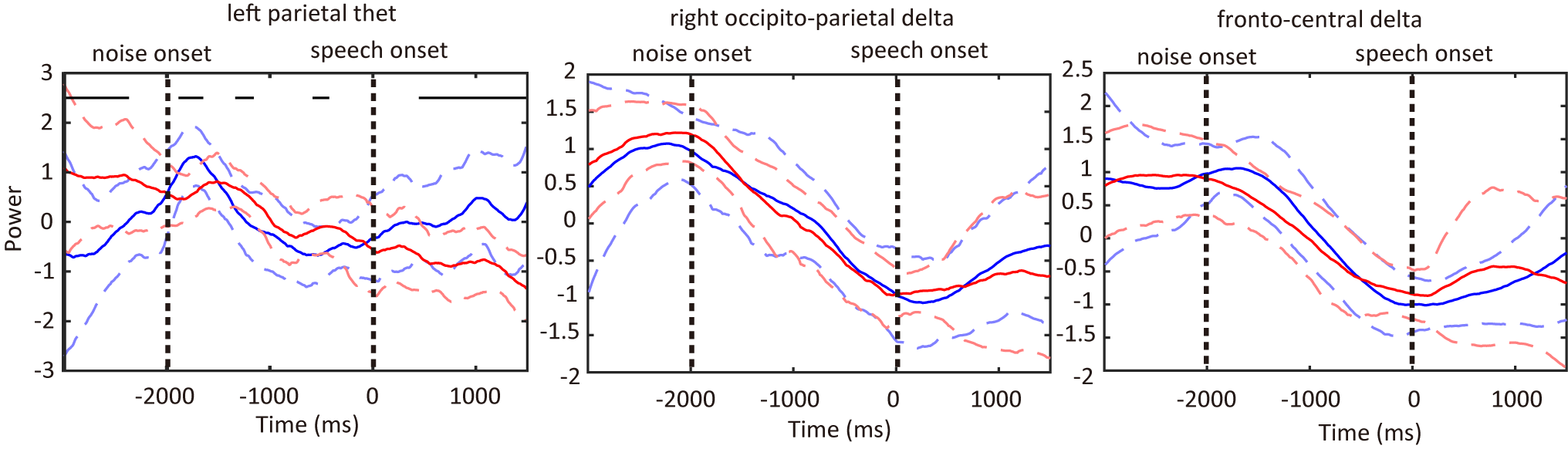

Supplement: S3 Fig — The EEG power for the EEG experiment (blue line) and the simultaneous EEG-fMRI experiment (red line). The power was averaged among the participants (mean±s.e.m.) for the left parietal theta (left), right occipito-parietal delta (middle), and fronto-central delta (right). The black horizontal bars on each panel represent the period where the significant difference was shown (p < 0.05). (TIF) [file pone.0183146.s003.tif]
